# Thymostimulin in advanced hepatocellular carcinoma: A phase II trial

**DOI:** 10.1186/1471-2407-8-72

**Published:** 2008-03-13

**Authors:** Matthias M Dollinger, Christa M Behrens, Joachim Lesske, Susanne Behl, Curd Behrmann, Wolfgang E Fleig

**Affiliations:** 1 First Department of Medicine, Martin-Luther-University Halle-Wittenberg, Ernst-Grube-Strasse 40, 06120 Halle, Germany; 2 Department of Medicine, Bad Reichenhall Hospital, Riedelstr. 5, 83435 Bad Reichenhall, Germany; 3 Department of Radiology, Martin-Luther-University Halle-Wittenberg, Ernst-Grube-Strasse 40, 06120 Halle, Germany; 4 University of Leipzig Hospitals and Clinics, Philipp-Rosenthal-Straße 27, 04103 Leipzig, Germany

## Abstract

**Background:**

Thymostimulin is a thymic peptide fraction with immune-mediated cytotoxicity against hepatocellular carcinoma *in vitro*. In a phase II trial, we investigated safety and efficacy including selection criteria for best response in advanced or metastasised hepatocellular carcinoma.

**Methods:**

44 patients (84 % male, median age 69 years) not suitable or refractory to conventional therapy received thymostimulin 75 mg subcutaneously five times per week for a median of 8.2 months until progression or complete response. 3/44 patients were secondarily accessible to local ablation or chemoembolisation. Primary endpoint was overall survival, secondary endpoint tumor response or progression-free survival. A multivariate Cox's regression model was used to identify variables affecting survival.

**Results:**

Median survival was 11.5 months (95% CI 7.9–15.0) with a 1-, 2- and 3-year survival of 50%, 23% and 9%. In the univariate analysis, a low Child-Pugh-score (p = 0.01), a low score in the Okuda- and CLIP-classification (p < 0.001) or a low AFP-level (p < 0.001) were associated with better survival, but not therapy modalities other than thymostimulin (p = 0.1) or signs of an invasive HCC phenotype such as vascular invasion (p = 0.3) and metastases (p = 0.1). The only variables independently related to survival in the Cox's regression model were Okuda stage and presence of liver cirrhosis (p < 0.01) as well as response to thymostimulin (p < 0.05). Of 39/44 patients evaluable for response, two obtained complete responses (one after concomitant radiofrequency ablation), five partial responses (objective response 18%), twenty-four stable disease (tumor control rate 79%) and eight progressed. Median progression-free survival was 6.4 months (95% CI 0.8–12). Grade 1 local reactions following injection were the only side effects.

**Conclusion:**

Outcome in our study rather depended on liver function and intrahepatic tumor growth (presence of liver cirrhosis and Okuda stage) in addition to response to thymostimulin, while an invasive HCC phenotype had no influence in the multivariate analysis. Thymostimulin could therefore be considered a safe and promising candidate for palliative treatment in a selected target population with advanced hepatocellular carcinoma, in particular as component of a multimodal therapy concept.

**Trial registration:**

Current Controlled Trials ISRCTN29319366.

## Background

While the incidence of hepatocellular carcinoma (HCC) is rising worldwide [1], only 25% of patients benefit from curative treatment and transarterial chemoembolisation (TACE) appears the only palliative therapy with proven benefit for intermediate stages of the tumor [2-4]. For patients with advanced or metastatic disease, no standard treatment has been established resulting in a life expectancy of less than 10% at 3 years. Systemic chemotherapy in particular has been disappointing, not only because of the chemoresistance of HCC, but because of major side-effects poorly tolerated by patients with liver cirrhosis.

Immunomodulation is a promising experimental strategy against HCC [5]. While interferon-based therapy has also been poorly tolerated with little benefit in clinical trials, thymostimulin showed an overall response rate of 24% associated with a significant increase in life expectancy in the only clinical phase II trial to date [6]. Moreover, virtually no side-effects were apparent. Isolated from calf thymus, thymostimulin is a standardized low-molecular protein fraction including thymosin-α1 and thymic humoral factor [7,8]. It has been shown to induce the proliferation and differentiation of T-lymphocytes and to stimulate the release of interferons and interleukin-2 [9]. *In vitro*, thymostimulin activates a selective dose-dependent cytotoxic reaction of Kupffer cells against HCC cell lines [10]. However, the antineoplastic effect *in vivo *and thus the patients likely profiting from therapy remain unclear.

This phase II trial was designed to substantiate the safety and efficacy of thymostimulin in the treatment of advanced HCC and identify clinical criteria to select patients benefiting from a randomized, controlled trial.

## Methods

### Eligibility

Patients with locally advanced or metastatic HCC not amenable to or failing established treatment were enrolled. Lesions were histologically proven or highly suspicious of HCC in two independent imaging techniques with elevated α-fetoprotein levels (AFP) over 400 ng/ml. Pretreatment of the HCC was allowed in case of tumor progress with the respective therapy; however, no treatment was to be given for at least 3 weeks prior to enrollment. Patients were required to be between 18 and 80 years of age and have an ECOG performance status of ≤ 3. Exclusion criteria were pregnancy/lactation, active second malignancy, severe concomitant disease (e.g. NYHA III-IV, serum creatinine level > 300 μmol/l) or severe decompensated liver function (bilirubin > 5 mg/dl, INR ≥ 2.3). None of the patients received anti-viral treatment with interferon. Ethical approval was obtained from the local ethical review board before study initiation and written informed consent from each patient before entering the study. The study was conducted in accordance with the ethical principles stated in the Declaration of Helsinki and the guidelines on good clinical practice.

### Study design

The study was designed as a prospective, uncontrolled and single-centre phase II trial, investigating effect and safety of thymostimulin in patients with advanced HCC. In case of tumor regress, secondary treatment of the HCC with loco-regional modalities was permitted. Primary endpoints of the study were overall survival as well as 1-, 2- and 3-year survival, secondary endpoints tumor response and progression-free survival according to standard WHO criteria, as well as toxicity according to ECOG criteria [11,12].

### Treatment

Thymostimulin is a licensed immunomodulating drug prepared from an extract of peptides from bovine thymus glands (Thymophysin CytoChemia^® ^25/50). Following removal of high-molecular cell components and proteins, the low-molecular active thymus peptides are isolated and standardized to a defined protein fraction. All patients received thymostimulin 75 mg subcutaneously for 5 days a week according to manufacturers specifications in addition to best supportive care as required. Treatment with thymostimulin was continued until one of the following criteria was met: disease progression, death of patient, unacceptable toxicity, patient refusal or incompliance, complete response for more than 5 months. Patients with tumor regress were allowed non-systemic concomitant treatment with radiofrequency thermal ablation (RFTA) or transarterial chemoembolisation (TACE), if the tumor was found to be accessible secondary to the study treatment. In case of tumor progress, patients were allowed to receive salvage therapy at the investigator's discretion.

### Pretreatment and follow-up evaluation

Pre-treatment and follow-up evaluation included a complete medical history, physical examination, blood count and chemistry as well as performance status. Cause, risk factors and extent of liver disease according to Child-Pugh status as well as prior treatment modalities were recorded at baseline. Tumors were assessed by abdominal ultrasound, chest X-ray and either dynamic computerized tomography (CT) or magnetic resonance imaging (MRI); Okuda- and CLIP-classifications were used for staging. Follow-up investigations were conducted at 6 and 12 weeks, and every three months thereafter until the end of the study. They also included survival data and documentation of concomitant therapies and toxicity of the medication. Tumor response was measured using abdominal ultrasound and CT or MRI scanning and evaluated according to WHO criteria by an experienced radiologist (C. Behrmann).

### Statistical methods

All analyses were by intention to treat. Comparisons of continuous variables were done by the Wilcoxon rank-sum method and for categorical variables by the Fisher's exact test. Survival time and progression-free survival were calculated from the start of therapy to the date of death or date of progression/death without progression, respectively. Surviving patients with a complete response were censored at the time of analysis. Survival curves were established with the Kaplan-Meier method, a stepwise forward Cox's regression analysis of survival was used to assess baseline predictors and the treatment effect simultaneously. The following variables were chosen for the univariate analysis: age, sex, weight, the presence of liver cirrhosis and Child classification, Okuda stage and CLIP score, AFP-level, multifocal tumor manifestation, ascites, vascular invasion, extrahepatic metastases, treatment with thymostimulin and treatment with other therapy modalities before or after study entry. Significant variables in the univariate analysis were introduced into the multivariate analysis. Calculations were done with the SPSS package (version 12.0.1).

## Results

### Patient characteristics

A total of 48 Caucasian patients were enrolled from July 2000 until September 2002. Four of the patients withdrew their consent before the first dose of thymostimulin was administered and their data had to be censored. The final study population consisted of 37 men and 7 women. Detailed demographic data and tumor-related characteristics are depicted in Table 1. Most patients had liver cirrhosis (84%) and 32% of participants suffered from deranged liver function at study entry (Child-Pugh-Score ≥ 7). Using the Okuda- and CLIP-classification, a majority of tumors were staged as intermediate HCC (Okuda stage II: 64%; CLIP 1–3 points: 79%); however, 32% showed signs of vascular invasion and 21% had extrahepatic metastases. About half of the patients (55%) had been treated prior to enrollment with surgical resection (R1 or R2 resection), RFTA (range 1–3 sessions), TACE (range 1–9 sessions) or systemic chemotherapy (mytomycin C, tamoxifen, retinoic acid or somatostatin), but suffered from tumor progression.

**Table 1:** Patient characteristics

**Characteristics (n = 44)**	
***Patients***	
Male/female, *n (%)*	37/7 (84/16)
Median age, *years (range)*	69 (41–80)
Median weight, *kg (range)*	74 (58–123)

***Origin of liver disease, n (%)***	
Alcohol abuse	20 (46)
HBV/HCV	8 (18)
NASH	5 (11)
Cryptogenic	11 (25)

***Tumor stage, n (%)***	
Liver cirrhosis	37 (84)
Child classification A/B/C	23/9/5 (52/21/11)
Okuda stage I/II/III	13/28/3 (29/64/7)
CLIP 0/1–3/4–6	2/35/7 (5/79/16)

***α-FP (ng/ml), n (%)***	
< 400	29 (66)
400- 10000	8 (18)
> 10000	7 (16)

***Tumor characteristics, n (%)***	
Ascites	13 (30)
Portal vein thrombosis	14 (32)
Multifocal tumor manifestation	38 (86)
Extrahepatic metastases	9 (21)

***Previous treatment, n (%)***	
*(combination possible)*	
Surgery	4 (9)
Radiofrequency ablation	5 (11)
Transarterial chemoembolisation	17 (39)
Systemic chemotherapy	4 (9)

### Treatment summary

At the time of analysis, all patients had stopped treatment with thymostimulin (Table 2) and 42 (96%) out of 44 patients had died, either because of tumor progression (n = 29/66%), hepatic failure (n = 10/23%) or causes unrelated to the HCC (n = 3/7%). The median follow-up was 11.8 months (range 0.4 to 56.8 months), the median length of treatment 8.2 months (range 0.4 to 54.3 months). 19 patients (43%) were solely treated with thymostimulin for the HCC with no other therapy modalities before or after study entry. Three patients (7%) showed a partial response to the study medication with tumors secondarily accessible to RFTA (1–2 sessions) or TACE (3 sessions). Two out of 44 patients received salvage therapy after tumor progression; one was treated with RFTA, the other died during an attempted hepatic resection. All patients were included in the intention-to-treat analysis of survival; however, 5 patients died within 6 weeks of enrollment before the first follow-up visit and their data were censored for the assessment of the treatment response.

**Table 2:** Reasons for termination of treatment and cause of death

	**Termination of treatment (n = 44)**
Death within 6 weeks of study entry^§^	5
Tumor progression	29
Hepatic failure with stable disease^§^	5
Other*	3
CR (one patient with concomitant RFTA)	2

* pneumonia after femur fracture, ventricular fibrillation and haemorrhage during surgical resection

^§ ^cause of death hepatic failure without proven tumor progress

### Overall survival

**Figure 1:**
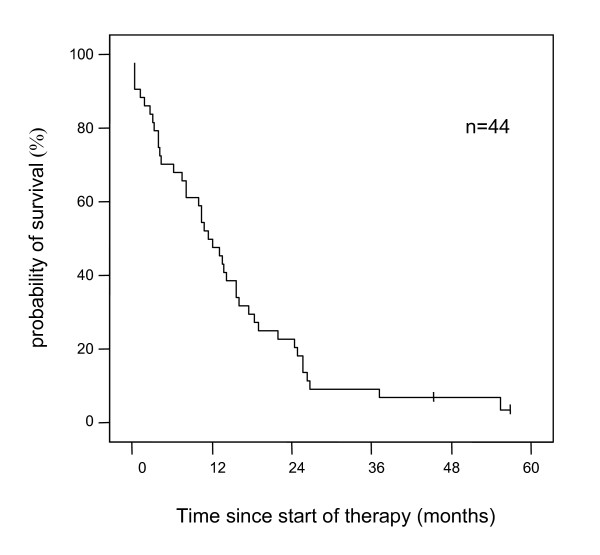
**Estimate of overall survival**. Kaplan-Meier graph showing probability of survival over time in percentage of patients treated (-- survival function, + censored).

**Figure 2:**
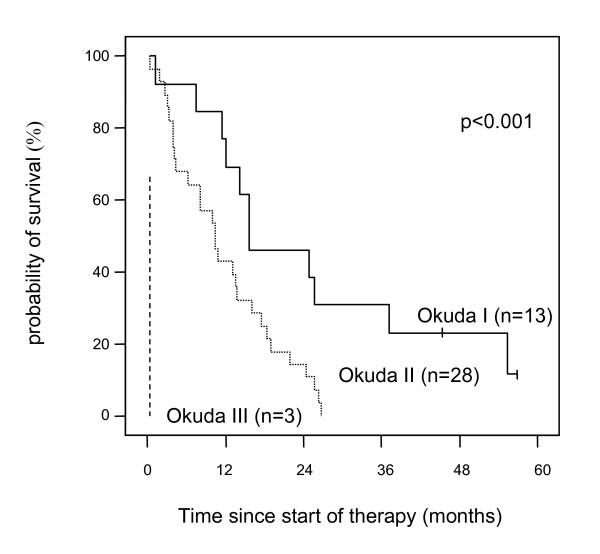
**Estimate of overall survival according to Okuda stage**. Kaplan-Meier graph showing probability of survival over time in percentage of patients treated and according to the Okuda stage of the hepatocellular carcinoma (-- survival function, + censored).

**Figure 3:**
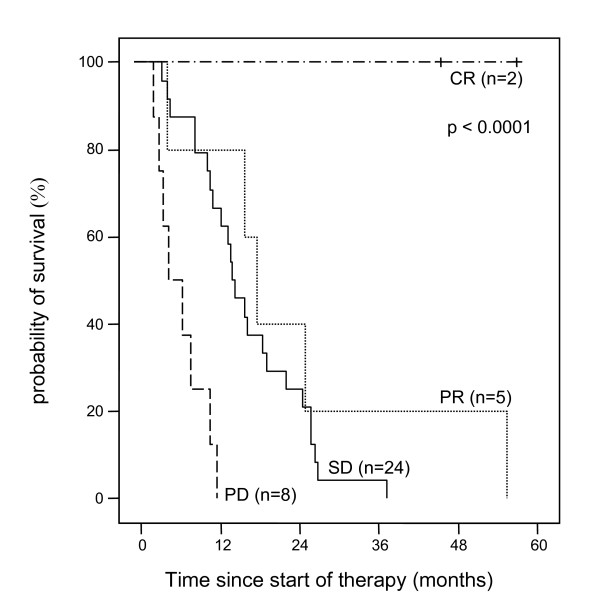
**Estimate of overall survival according to response to study treatment**. Kaplan-Meier graph showing probability of survival over time in percentage of patients treated and according to the response to the study treatment with thymostimulin (-- survival function, + censored).

The median survival for all patients started on thymostimulin was 11.5 months (95% CI 7.9–15.0) with two patients remaining alive at the time of analysis (Figure 1). The probabilities of survival at 1, 2 and 3 years were 50%, 23% and 9%, respectively. Patients solely treated with thymostimulin for the HCC had a median survival of 10.1 months (95% CI 0.9–19.3) with a probability of survival at 1, 2 and 3 years of 32%, 16% and 0%. The effect of baseline predictors and the treatment on survival of the patients was assessed using a uni- and multivariate analysis. Thus, a low Child-Pugh-score, a low score in the Okuda- and CLIP-classification or a low AFP-level at study entry were associated with better survival (Table 3). In contrast, treatment with therapy modalities other than thymostimulin before or after study entry or signs of an invasive HCC phenotype such as vascular invasion or extrahepatic metastases had no significant impact on survival. The only variables independently related to survival in the Cox's regression model were Okuda stage (Figure 2) and presence of liver cirrhosis at baseline (negative correlation, p < 0.01) as well as the response to treatment (positive correlation, p < 0.05; Figure 3).

**Table 3:** Variables associated with overall survival in the univariate analysis

**Variables**	**No. of patients**	**Median survival *(months ± SD)***	**p-value**
Overall	44 (100%)	11.5 ± 1.8	

No cirrhosis/Child A	30 (68%)	13.7 ± 1.7	
Child B	9 (21%)	6.3 ± 2.8	p = 0.01
Child C	5 (11%)	0.4 ± 0.1	

Okuda stage I	13 (29%)	15.7 ± 6.4	
Okuda stage II	28 (64%)	10.4 ± 1.7	p < 0.001
Okuda stage III	3 (7%)	0.4 ± 0.0	

CLIP 0	2	37.1*	
CLIP 1	10	13.7 ± 2.7	
CLIP 2	15	13.6 ± 2.7	p < 0.001
CLIP 3	10	8.1 ± 1.6	
CLIP 4–6	7	0.5 ± 0.1	

AFP < 400 ng/ml	29 (66%)	16.2 ± 3.5	p < 0.001
AFP > 400 ng/ml	15 (34%)	6.3 ± 2.6	

thymostimulin only	19 (43%)	10.1 ± 4.7	p = 0.1
additional therapy modalities	25 (57%)	13.7 ± 2.1	

no vascular invasion	30 (68%)	11.5 ± 2.2	p = 0.3
with vascular invasion	14 (32%)	10.8 ± 2.8	

no extrahepatic metastases	35 (79%)	13 ± 1.7	p = 0.1
with extrahepatic metastases	9 (21%)	8 ± 7.5	

* SD not applicable due to low patient number

### Response and progression-free survival

39 (88.6%) out of 44 patients were analysable for treatment response. Response rates and the associated median survival are depicted in Table 4. Two patients achieved a complete response and were alive at the time of analysis (survival time 45.3 and 56.8 months, respectively). In the first patient, the tumor became secondarily accessible to local ablation after 10 months of treatment with thymostimulin. He received concomitant therapy with 2 sessions of RFTA as stipulated in the protocol. 5 months later thymostimulin was stopped and the patient remained free of tumor until the time of analysis (treatment response 28 months). The second patient achieved a complete response without concomitant therapy after 8 months of treatment with thymostimulin. 5 months later, the study medication was stopped and the patient remained free of tumor for 11 months until tumor recurrence necessitating further treatment outside of the study. Another 8 patients in the study survived longer than 2 years. Of these, two showed a partial response to thymostimulin, in one rendering the tumor secondarily accessible to RFTA (survival time 24.8 and 55.4 months, respectively). Further 6 patients achieved stable disease with the study medication (survival time 24.4 to 37.1 months). All patients died of tumor progress except for one, who died during an attempted rescue therapy by hepatic resection after tumor progress.

**Table 4:** Tumor response and associated median survival (n = 39)

	**No. of patients**	**Median survival *(range)***	**95% CI**
Complete response (CR)	2 (5%)	> 51 months*	
with concomitant treatment (RFTA)	1	(> 51 months*)	
without concomitant treatment	1	(> 51 months*)	

Partial response (PR)	5 (13%)	18 months	14–22
with concomitant treatment (TACE/RFTA)	2	(range 15.7–55.4)	
without concomitant treatment	3	(range 3.9–24.8)	

Stable disease (SD)	24 (61%)	14 months	11–17
Progressive disease (PD)	8 (21%)	4 months	0 – 8
Objective response (CR+PR)	7 (18%)	25 months	20–29
Tumor control rate (CR+PR+SD)	31 (79%)	16 months	11–20

* patients alive at time of analysis

**Figure 4:**
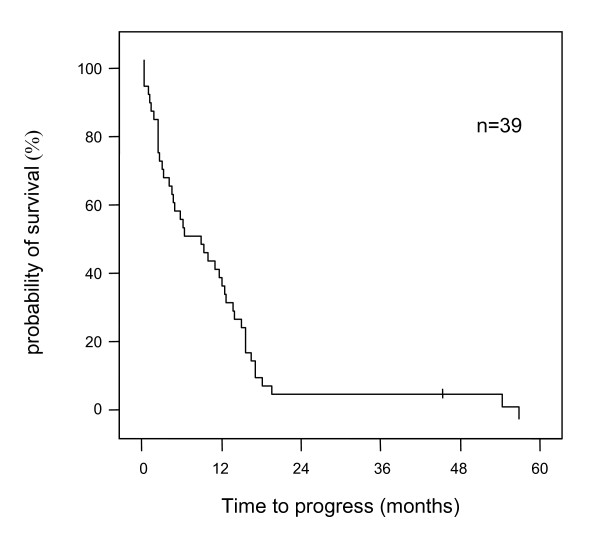
**Estimate of progression-free survival**. Kaplan-Meier graph showing probability of progression-free survival over time in percentage of patients treated (-- survival function, + censored).

The median progression-free survival was 6.4 months (95% CI 0.8–12; Figure 4) and was dependent on the score in the Okuda- (p < 0.001) and CLIP-classification (p < 0.01), the AFP-level (p < 0.001) and the treatment with other therapy modalities than thymostimulin before or after study entry (p < 0.01).

### Toxicity

None of the patients suffered from severe adverse events during therapy. Ten of the patients (23%) reported local erythema, itching or pain at the injection site (grade I on ECOG common toxicity criteria). No patient had to reduce or stop the study medication or had to be admitted to the hospital due to side effects.

## Discussion

While TACE has become the palliative treatment of choice for HCC in patients with preserved liver function and tumors limited to the liver parenchyma [13,14], there is no standard treatment for HCC exceeding these criteria or failing conventional therapy. In our phase II study, we used the immunomodulatory compound thymostimulin, a standardized thymic peptide fraction, to treat patients with advanced HCC not suitable or refractory to therapy options such as resection, RFTA or TACE. A similar phase II study was published in 1996 on 46 patients with advanced HCC not eligible for surgery, reporting an overall tumor response rate of 24% and a tumor control rate of 63% [6]. 13% of patients obtained a complete response with a median duration of 19 months and a median survival of 27 months compared with 4 months in the 37% of patients with progressive disease. Despite the promising results however, the publication missed out criteria to distinguish between responders and non-responders to thymostimulin and thus to select patients who would likely benefit from treatment.

Similar results were obtained in our study using thymostimulin with a one- and two-year-survival better than anticipated by Okuda [15] or the Clip Study Group [16], bearing in mind the selection of our patients not suitable or refractory to conventional therapy (Table 5). Recently, the Barcelona Clinic Liver Cancer (BCLC) staging classification was established proposing treatment options and survival probabilities for early (A), intermediate (B), advanced (C) and terminal (D) stage HCC [17]. Data for stage C and D of the disease were in part based on an analysis of the combined control arms of a chemoembolisation and a tamoxifen trial, thus providing the natural history of untreated HCC [18]. While patients in group C still had a 1-, 2- and 3-year survival of 29%, 16% and 8%, respectively, patients in group D had a life expectancy of less than 6 months. Comparisons with our data are complicated by the fact that our study population included patients with both, BCLC stage C and D of the disease. However, an effect of thymostimulin over best supportive care will only be proven in a randomized controlled trial, highlighted recently by the failure of other alternative treatment options such as doxorubicin, tamoxifen or octreotide in meta-analyses or phase III studies [19-21]. The only possible new therapy for advanced HCC with a survival benefit over best supportive care in a large controlled trial is the protein kinase inhibitor sorafenib, presented to date only in a meeting abstract [4,22]. Its effect, however, has only been proven for selected patients with well-preserved liver function (not more than 5–6 Child Pugh points) and at the expense of common side-effects in up to 78% of cases [23,24].

**Table 5:** Patient survival overall and by Okuda stage and CLIP score

	**Thymostimulin (present data)**		**Okuda et al. 1985 [16]**		**CLIP 2000 [17]**	
	**1-year survival**	**2-year survival**	**1-year survival**	**2-year survival**	**1-year survival**	**2-year survival**

Overall	50 %	23 %	26 %	15 %	48 %	28 %
Okuda stage						
I	69 %	38 %	49 %	32 %	68 %	48 %
II	40 %	11 %	18 %	9 %	36 %	13 %
III	0 %*	0 %*	3 %	3 %	21 %	10 %
CLIP score						
0	100 %*	100 %*			84 %	65 %
1	70 %	40 %			66 %	45 %
2	60 %	20 %			45 %	17 %
3	30 %	10 %			36 %	12 %
4–6	14 %*	0 %*			9 %	0 %

* small case number

Interestingly, the Barcelona data showed cancer-related symptoms and an invasive HCC phenotype with vascular invasion or extrahepatic spread to be the best predictors of outcome for intermediate and advanced tumors [18]. In contrast, outcome in our study rather depended on liver function and intrahepatic tumor growth (presence of liver cirrhosis and Okuda stage) in addition to response to thymostimulin, while an invasive or metastatic HCC phenotype had no influence in the multivariate analysis. It may thus be speculated, that the immunmodulatory effect of thymostimulin requires a functioning immune system. All postulated antineoplastic pathways of thymostimulin – stimulation of T lymphocytes to release interleukin-2 and interferons or activation of Kupffer and Natural Killer cells with release of tumor necrosis factor-α [9,10] – are impeded by a deteriorating liver function [25-27]. Indeed, HCC growth itself has been linked to a depressed immune function in patients with liver cirrhosis [28]. Thus, the therapeutic impact of thymostimulin appears also to depend on a preserved liver function and a limited intrahepatic tumor size.

An effect of the treatment modalities prior or in addition to thymostimulin has to be assumed in our study. Even though the response to thymostimulin only, not treatment with other modalities, was selected as a prognostic factor in the multivariate analysis of overall survival, progression of the tumor was dependent on a multimodal treatment. Obviously, thymostimulin is solely a palliative treatment of HCC, although with a reasonable tumor response rate (18%) and very good tumor growth control (79%) in the present series compared with conventional chemotherapy [14]. Since virtually no side-effects were evident in this and the previous phase II study [6], it might well be a suitable immunomodulatory component of a multimodal antineoplastic therapy [29-31].

## Conclusion

In conclusion, this phase II study confirms the previous report on the potential efficacy and excellent safety profile of thymostimulin in the treatment of HCC. As palliative treatment, a controlled trial is required to unequivocally demonstrate the superiority of thymostimulin over best supportive care. Selection of the target population appears to be necessary regarding liver function and intrahepatic tumor growth, while an invasive or metastatic HCC phenotype has no impact on tumor response. Thus, thymostimulin might be a suitable and well-tolerated component of a multimodal therapy concept for advanced HCC, in particular in combination with local ablative strategies.

## Abbreviations

CI: Confidence interval; HCC: Hepatocellular carcinoma; TACE: transarterial chemoembolisation; RFTA: radiofrequency thermal ablation; AFP: α-fetoprotein; CT: computerized tomography; MRI: magnetic resonance imaging, ECOG: Eastern Cooperative Oncology Group; WHO: World Health Organization.

## Competing interests

The author(s) declare that they have no competing interests.

## Authors' contributions

MMD analyzed the data, drafted and finalized the manuscript, and coordinated its submission. CMB and LJ enrolled patients in the clinical protocol. SB performed the statistical analysis. CB evaluated the CT and MRI scans. WEF conceived the study and was the primary investigator. All authors read and approved the final manuscript.

## Pre-publication history

The pre-publication history for this paper can be accessed here:


